# Simulating the Genetics Clinic of the Future — whether undergoing whole-genome sequencing shapes professional attitudes

**DOI:** 10.1007/s12687-021-00561-0

**Published:** 2022-01-27

**Authors:** Minna Brunfeldt, Harriet Teare, Daan Schuurbiers, Daniela Steinberger, Elianne Gerrits, Marleena Vornanen, Nine Knoers, Helena Kääriäinen, Terry Vrijenhoek

**Affiliations:** 1 Finnish Institute for Health and Welfare, Mannerheimintie 166, Helsinki, Finland; 2 Nuffield Department of Population Health, University of Oxford, Oxford, UK; 3 De Proeffabriek, Arnhem, The Netherlands; 4 Institute of Human Genetics, Justus Liebig University, Giessen, Germany; 5 Department of Genetics, University Medical Centre Utrecht, Utrecht, The Netherlands; 6 University of Helsinki, Helsinki, Finland; 7 Department of Genetics, University Medical Centre Groningen, Groningen, The Netherlands

**Keywords:** Whole-genome sequencing (WGS), Genetic testing, Genomic data, Healthcare

## Abstract

Whole-genome sequencing (WGS) can provide valuable health insight for research participants or patients. Opportunities to be sequenced are increasing as direct-to-consumer (DTC) testing becomes more prevalent, but it is still fairly unusual to have been sequenced. We offered WGS to fourteen professionals with pre-existing familiarity with an interest in human genetics — healthcare, science, policy and art. Participants received a hard drive containing their personal sequence data files (.BAM,.gvcf), without further explanation or obligation, to consider how experiencing WGS firsthand might influence their professional attitudes. We performed semi-structured pre- and post-sequencing interviews with each participant to identify key themes that they raised after being sequenced. To evaluate how their experience of the procedure evolved over time, we also conducted a questionnaire to gather their views 3 years after receiving their genomic data. Participants were generally satisfied with the experience (all 14 participants would choose to participate again). They mostly decided to participate out of curiosity (personal) and to learn from the experience (professional). Whereas most participants slightly developed their original perspective on genetic data, a small selection of them radically changed their views over the course of the project. We conclude that personal experience of sequencing provides an interesting alternative perspective for experts involved in leading, planning, implementing or researching genome sequencing services. Moreover, the personal experience may provide professionals with a better understanding of the challenges visitors of the Genetics Clinic of the Future may face.

**Supplementary Information:**

The online version contains supplementary material available at 10.1007/s12687-021-00561-0.

## Introduction

The complexities associated with whole-genome sequencing (WGS) and its introduction into clinical care (van El et al. 2013; Berg et al. 2011; Vears et al. 2018) have evoked extensive discussions among professionals involved in genetics. Some studies have provided information about the experiences of — supposedly healthy — participants in beyond-the-clinic WGS projects (Ball et al. 2014; Beck et al. 2018; Reuter et al. 2018). We suggest that these discussions could benefit from the perspectives of professionals that have firsthand experience of WGS, having undergone sequencing themselves, and that this could be particularly insightful if their perspectives are gathered before and after sequencing, noting any changes in their opinions.

The attribution of personal experience to professional behaviour is an emerging theme in health policy research. It has been shown how having a disability shapes students’ and clinicians’ interactions with their patients (Battalova et al. 2020), and how doctors who become patients change their views towards risks and benefits of treatments (Klitzman 2006). Simultaneously, there is an emerging culture of empowering individuals to be the creators of solutions, sometimes unconventional, to challenges they encounter (‘Maker movement’) (Awori and Lee 2017). In general, the interest for adding personal perspectives to innovation trajectories is increasing across healthcare domains.

Rapid developments in genomic sequencing technologies have raised expectations for increased use of DNA sequencing in the clinic. Currently, sequencing is primarily used as a diagnostic tool for rare diseases or genetic predispositions (e.g. familial cancers). With recent technological advances, especially the breakthrough of next-generation sequencing (NGS), our understanding of human genotypes that are involved in disease has increased enormously. During the recent years, NGS has become faster, cheaper and more accurate (Stark et al. 2019; Machini et al. 2019; Veltman et al. 2013). Expanded use of WGS and whole-exome sequencing (WES) will improve diagnostics of rare genetic disorders, personalise treatment to suit specific patients, and anticipate strategies for prevention. This provides opportunities to shift the focus from disease-treatment, to more personalised and preventive care. While benefits for the community are not established yet, routine implementation of sequencing in healthcare faces considerable challenges that go far beyond the sequencing technologies per se. The potential of WES and especially WGS for research and diagnostics depends on solutions to major challenges around data sharing and control, informed consent, and the role of genome data within and beyond the clinic (Veltman et al. 2013; Leitsalu et al. 2016; Lange et al. 2020; Brunfeldt et al. 2018). Since these challenges cross disciplinary boundaries, viable solutions will depend on profound understanding of them among the range of disciplines involved (Lange et al. 2020).

At the same time, a growing number of people obtain genomic information outside the clinic. Individual citizens may get WGS data via various research projects, including biobanks, or may buy WGS from direct-to-consumer (DTC) companies. There are no reliable estimations on the numbers of WGS investigations performed at present nor how often the full data is shared with those tested. We have studied the approach taken by BBMRI-ERIC biobanks and most of them were planning to return some data to their donors but had not done so yet (Brunfeldt et al. 2018). Most DTC companies use genome-wide association studies (GWAS) but some of them also offer WGS. In these situations, users may strive to interpret their data with the help of bioinformatics companies or tools that have been developed for self-interpretation of genomics (e.g. Promethease) (Promethease 2021).

While the potential interest in using genomic data from people’s personal domains for clinical decision making is still unclear, it is likely that pressure will grow on the healthcare system to make some use of these data. There are already plenty of examples of data being usefully contributed by patients, for example the growing reliance on Patient Report Outcomes Measures (Beard et al. 2015) and research studies that acknowledge the value of the experience that patients provide (Teare et al. 2017; Rosa et al. 2015). Alongside this, the increase of websites offering symptom tracking, apps measuring biometrics, and wearables gathering health-related real-time data, demonstrates a shift in the traditional patient-doctor interaction (Jandoo 2020). PatientsLikeMe website allows patients to share their experiences with other patients, and it has become a valuable research resource (Wicks et al. 2011).

Here, we transpose the paradigm of personal experience contributing to professional attitude to the genetics field, and explore whether personal experience affects professional perspectives on WGS. As part of the Horizon 2020-funded Genetics Clinic of the Future (GCOF) project, we provided consortium members with the opportunity to have their own genome sequenced. The GCOF was a multidisciplinary EU-funded project (2015–2017) that aimed to develop ideas and tools for effective, patient-driven, and sustainable use of genomic data in future healthcare. Experiencing the process of personally undergoing WGS could extend participants’ knowledge and understanding beyond theoretical considerations, gaining first-hand experience with what it means to have and use such data. Participants explored the consent process, the technological feasibility as well as the personal, societal and clinical consequences of using genomic information.

The objectives of the project were (1) to study how experts (involved in leading, planning, implementing, recommending, communicating and researching genome sequencing services) experience having their genome sequenced, (2) monitor and assess pre-sequencing expectations of genomic information and to what extent these expectations are met post-sequence and (3) to explore the influence on receiving personal genomic data to professional attitudes.

## Materials and methods

We performed WGS on 14 representatives from consortium partners who may represent potential stakeholders in the Genetics Clinic of the Future. We provided participants with their WGS data, and interviewed them before and after receipt of the data. Additionally, we implemented a brief follow-up survey 3 years after sequencing to understand longer-term responses.

### The participants

**Fig. 1:**
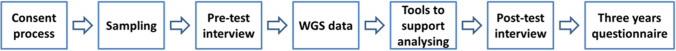
Project flow chart representing the phases of the project

In the mid-term meeting, the project coordinator indicated that WGS would be available to one representative from each of the consortium partners, entirely voluntarily. The representatives could either volunteer themselves or extend the offer to one of their colleagues. The participants were from several countries, represented universities, patient organisations, companies involved into genetics and had a wide variety of disciplines. The implications of sequencing were openly discussed, with ethical issues relating to being both partners and participants discussed at length. Sequencing was not included as a formal deliverable of the project, to avoid any worries of coercion. The participants could carefully consider their involvement, and to withdraw at any time from the simulation project (project flow chart is presented in Fig. 1).

### Ethics statement

All participants consented for data collection and publication. The Medical Research Ethics Committee (MREC) Utrecht confirmed that the Medical Research Involving Human Subject Act (WMO) does not apply to this study and that therefore formal approval was not required (reference number WAG/mb/16/014283) (Appendix 1).

### Consent

An accredited or broadly accepted consent procedure for explorative WGS on apparently healthy individuals was not available. Therefore, we co-created a custom consent procedure with all consortium partners, consisting of an information sheet (Appendix 2) and a consent form (Appendix 3). The consent form was an adapted version of common consent forms for clinical sequencing, with additions on the ownership. In addition to the information sheet, the process and consequences of the study were deeply discussed, and the participants were encouraged to ask questions.

### Sequencing and the process of delivering genomic data to participants

The department of the UMC Utrecht provided saliva collection kits. The UMC Utrecht isolated DNA from the saliva samples and used this for sequencing on an Illumina HiSeq X Ten system, according to standard operating procedures. The raw (.fasta), mapped (.bam) and annotated (.vcf) files were stored on hard disks and personally delivered to each participant. The data files were stored on hard disks and personally delivered to each participant. After delivery, all files were deleted from the UMC Utrecht servers which made each participant the sole possessor of his/her genome data. Initially, the participants received no support on how to analyse the data unless they could arrange this themselves. Three months after the participants had received their genome data, bio.logis offered them a personal genomics module for selected clinical questions concerning pharmacogenetics and carrier screening for common recessively inherited diseases. They were also alerted to existing tools that might help examine the data in more detail, including DNA.Land, Open Humans program, literature retrieval system Promethease, Integrated Genomics Viewer (IGV) and direct-to-consumer service 23andMe. In addition, bio.logis and Cartagenia (which has since become Agilent, Alissa) services were offered to interpret the genomic data more extensively.

### Interviews

We performed two interviews with all participants: (1) Pre-test interviews were held before the participants had received their genomic data (between August and October 2016; average duration 30 min per interview, range 16–51 min) (Appendix 4), and (2) Post-test interviews were held, approximately 3 months after receipt of the data and after receiving the bio.logis personal genomics report (between May and June 2017; average duration of 31 min per interview, range 19–54 min) **(**Appendix 5). Both the pre- and post-test interviews were semi-structured, based on a protocol that had been drafted and internally validated by a group of GCOF participants. Interviews were conducted via Skype, and were voice-recorded and transcribed. All interviews were undertaken by a single researcher (MB) to achieve the best possible consistency.

### Questionnaire

In the beginning of 2020 — 3 years after the data were handed over to participants — we invited all participants to respond to an internally validated questionnaire, using Webropol Tool (Appendix 6). The aim was to figure out if the participants had returned to their genomic data afterwards and if their thoughts about the experience had remained the same or possibly changed.

### Data analysis

The interviews were transcribed verbatim. We performed iterative coding on the transcripts of the interviews to identify key issues that participants raised in response to being sequenced. Based on the interview transcripts, we composed a summary of each participant’s replies for an overall picture on the content of the interviews and to ease the data handling. The transcripts were not returned to the participants for comments and/or corrections, and the participants did not provide feedback on the findings. We did not provide subject numbers for quotation, because of the data privacy, since there were only a small number of participants.

The data derived from the follow-up questionnaire was linked to the interview data question-specifically for comparison and for following the personal pathways. In the data analysis, we pointed out the events that expressed how the participants experienced the process.

## Results

Fourteen genetics-affiliated professionals from organisations involved in the GCOF project had their genome sequenced. Most participants (11/14) were consortium partners in the GCOF project, while the others were volunteers from institutes represented within the project (3/14). Participants were born between 1962 and 1990. They were from 10 different European countries and were predominantly male (9/14). Participants were generally highly educated (B.Sc. or higher) and professionally active in disciplines with varying affiliation to genetics: genomics research, clinical genetics, bioinformatics, ethics, social science, patient organisations and art. Three participants had previous experience with personal genomic testing, either in research setting or via DTC services.

In correspondence with objectives, the interviews and the brief questionnaire provided insight into (1) the experience of being sequenced, (2) how the pre-sequencing expectations were met in the light of the post-sequence interview and (3) the influence on professional attitudes.

### Experience

Participants generally perceived the overall experience as positive; all (14/14) mentioned that they would choose to participate again and confirmed that they were satisfied with the consent process. Participants’ motivation for participation was often a mixture of professional interest and personal curiosity. When asked what they were planning to do with the data, participant responses varied considerably. Also, many participants, at least at first, indicated they were not yet sure what kind of information they would glean from the data (Box 1).
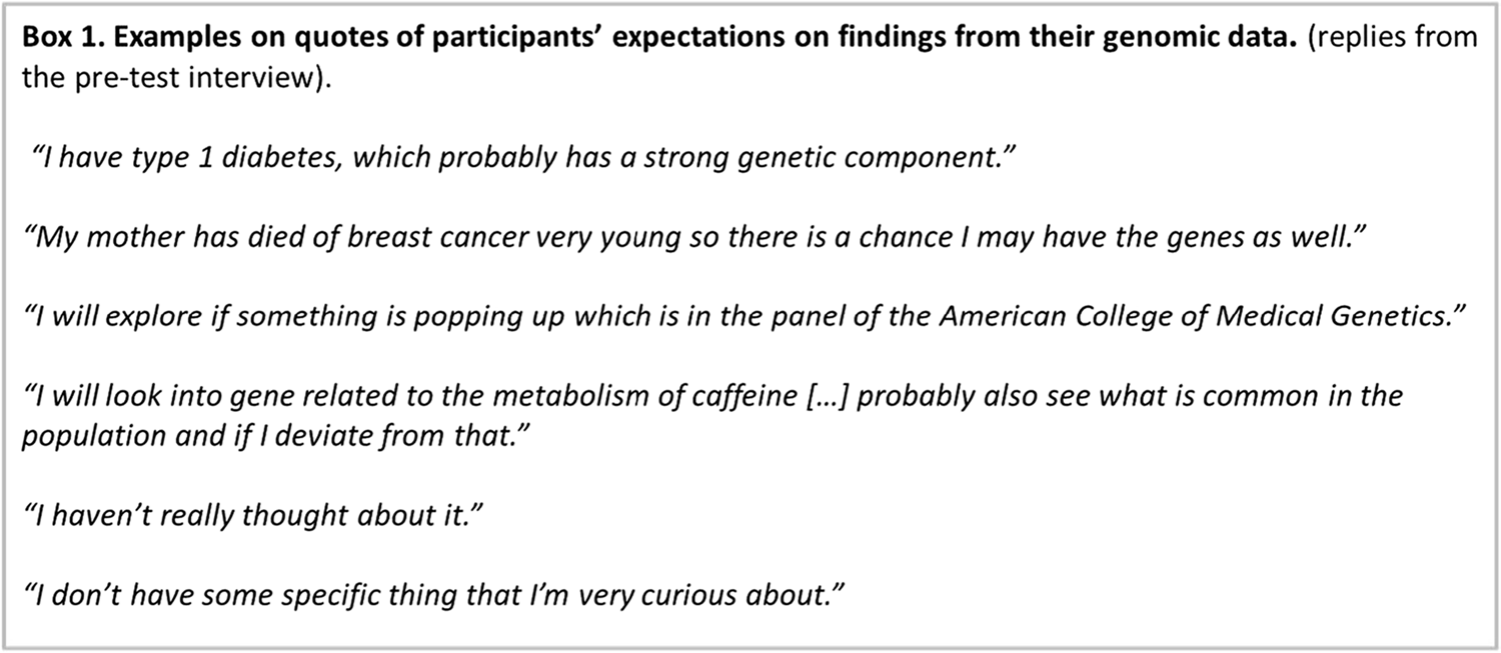


While participants were generally satisfied, they also expressed some worries, doubts and difficulties (Box 2). Some participants were anxious concerning their own health and the risk of finding something unpleasant or alarming, e.g. cancer risk variants. Others were more worried about the consequences for their family members, e.g. children. In addition, there were concerns about data privacy, e.g. companies using the data or data stealing (Box 2).
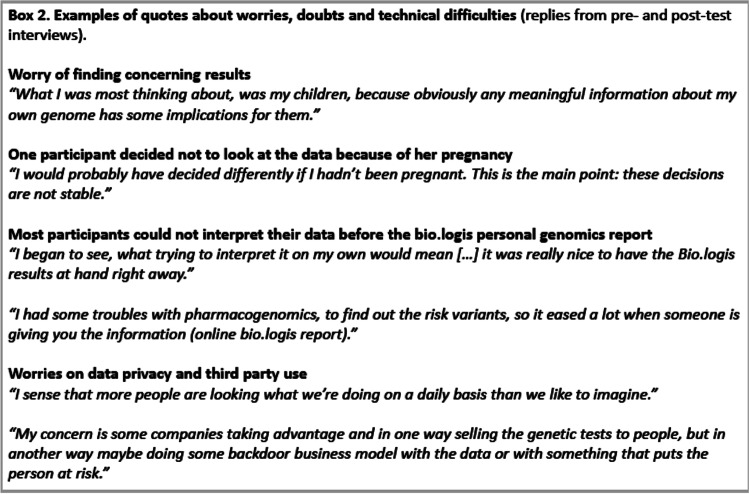


Most participants (10/14) had technical difficulties accessing the raw data. As non-professionals in informatics, they lacked the necessary infrastructure and tools to access and/or analyse the ± 60 Gb.BAM files, and were unfamiliar with commonly used file formats for genome data (e.g..fasta,.bam,.vcf). The responses to these challenges were varying; participants turned to the project coordinator, to colleagues, got frustrated, or even gave up accessing the data altogether. Of the four participants who had no trouble accessing the data, two were able to work with the data entirely by themselves based on their prior experience. The online reports displaying individual results (for example, pharmacogenomics) for each participant from the bio.logis’ personal genomics portal (3 months after reception of the raw data) provided all participants with at least some understandable results.

### Post-test interview insight into pre-sequencing plans and expectations

**Fig. 2:**
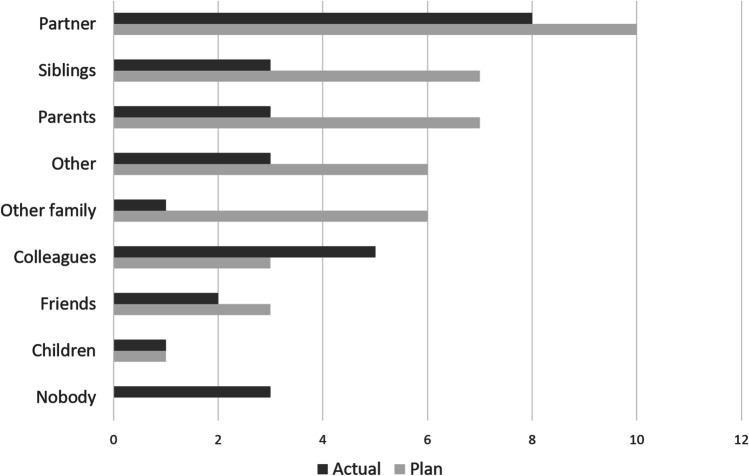
Transparency about sequencing results

How open participants were about their genomic results (Fig. 2) varied considerably. In general, participants considered their close family (partner, siblings, and parents) as the most important people to share results with. Still, there was a considerable discrepancy between the *intention* to share results and actually doing it (Fig. 2). For instance, only three of the 7 participants who indicated a plan to share results with parents or siblings eventually did so. One participant waived the self-imposed principle to keep it a secret in order to be able to securely and sustainably archive the data; the other ultimately recognized the value of the experience in discussion with colleagues. Of the participants, 8/14 had offspring, of which a few mentioned by their own initiative that their children were too young to understand the results and therefore did not share the results with them. The participants generally overestimated the number of people they would share their genomic results with.

**Fig. 3:**
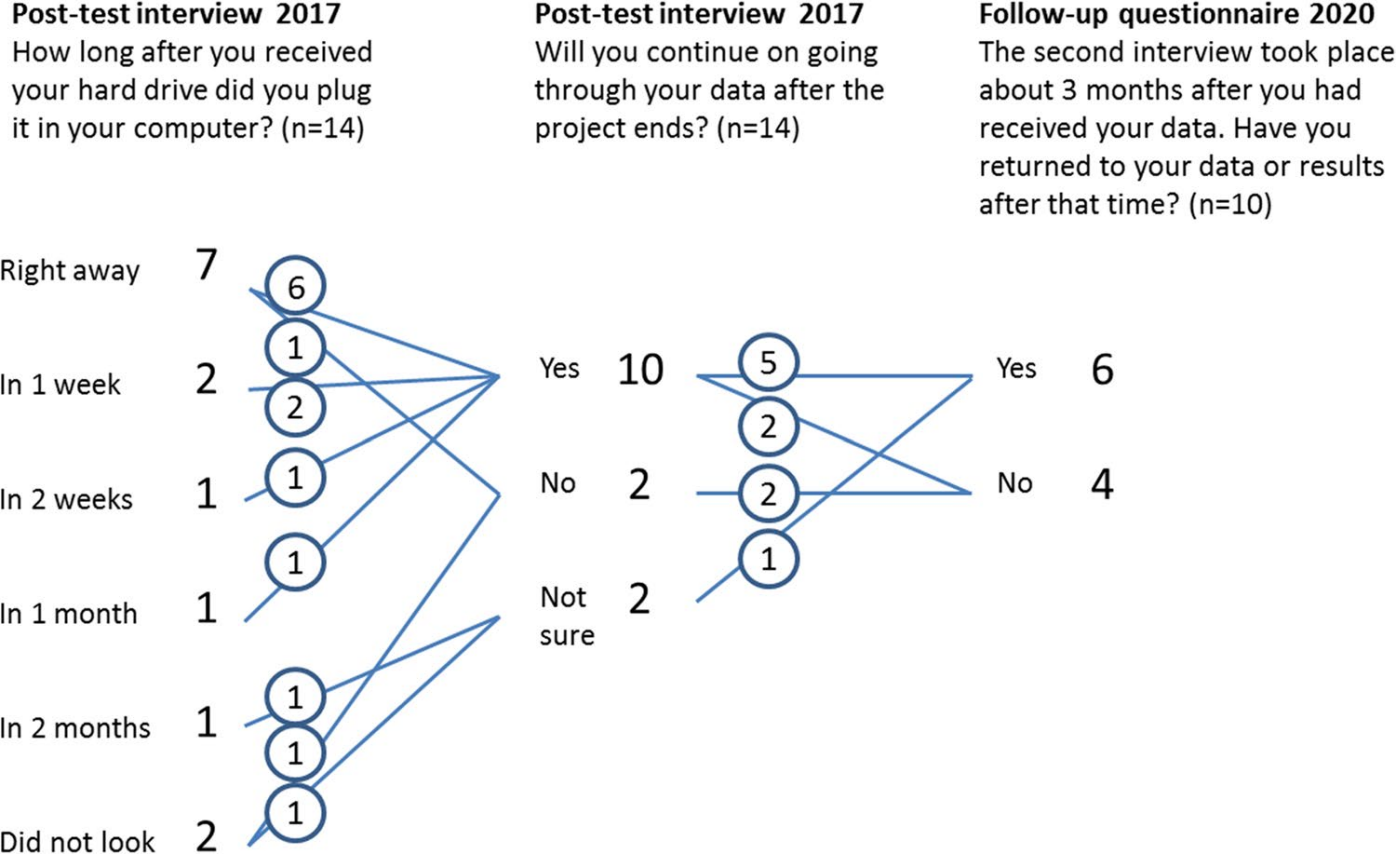
Personal pathways concerning how fast the participants plugged the genomic raw data hard drive to computer and if they continued surveying the data after

**Fig. 4:**
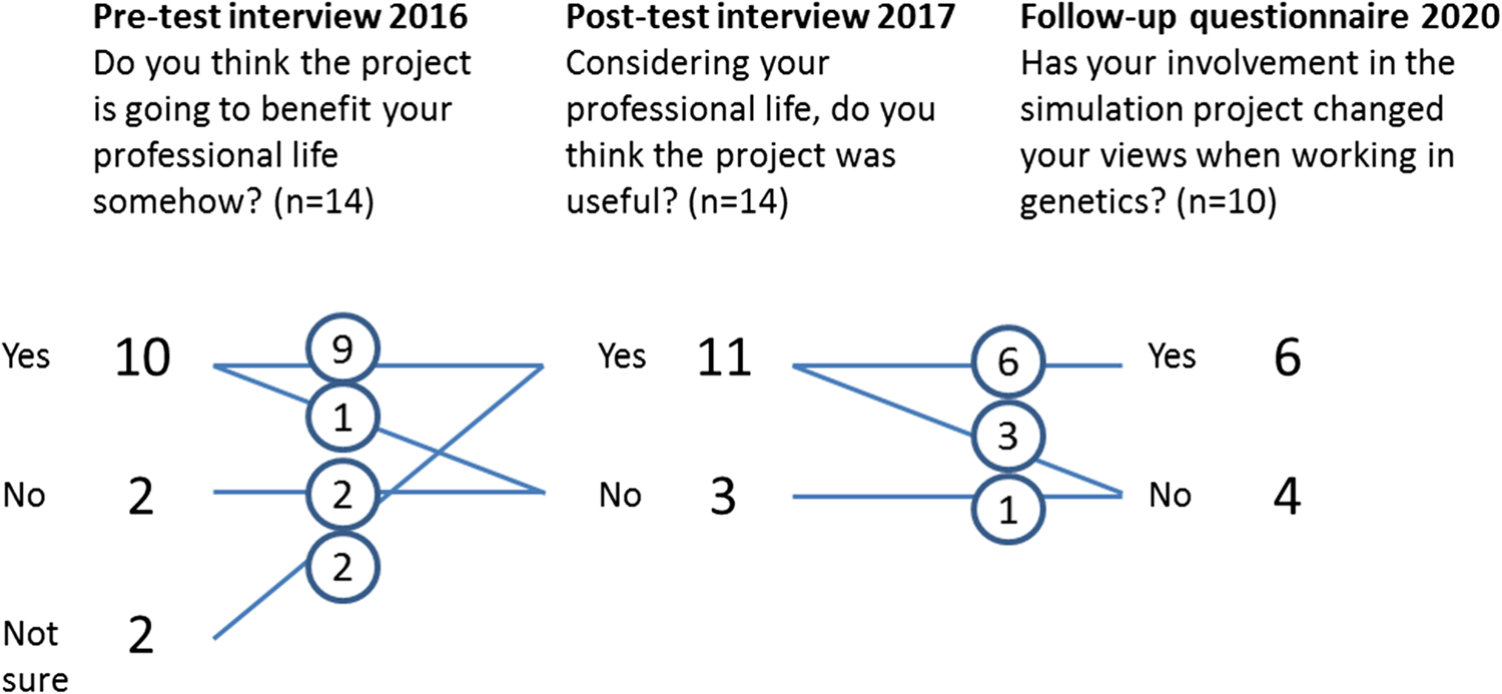
Timeline on how the participants experienced the participation to the project benefited their professional life

Half of the participants (7/14) accessed their data files immediately after receiving the hard drive. Later, 11 participants had opened their raw genomic data files and had also viewed their bio.logis personal genomics report. On the other hand, three participants consciously decided not to pursue analysis of their own data, or at least not all of it. One became pregnant between the decision to participate and the moment she received the data, and therefore made a personal choice not to look at either the WGS data or the bio.logis report. One person had not looked at the WGS data by the time of the post-test interview but had briefly looked at the bio.logis report. One person did open the files immediately, but had technical problems because of the data format and also felt worried about the data privacy issues and did not return to the data (Fig. 3). Three years after having received the data, we reached 10/14 participants for a brief follow-up survey. Most (6/10) participants indicated having returned to their data or results at least a few times (Fig. 3). Of them 3/6 replied that they had not changed their health behaviour, while 3/6 reported having made changes to avoid (statins in the future) or stop (ibuprofen, oral contraceptives) certain medications. All belonging to this group (6/6), replied they had not been worried because of the results. All 10/10 participants had kept the data stored and had not deleted it (Fig. 4).

The participants referred to pharmacogenomics results as the most interesting information. They also mentioned other results of interest, such as carrier states, disease variants in family, nutrition related variants and also variants relating to gluten sensitivity, celiac disease, lactose intolerance and diabetes. One participant reported having been identified as a carrier of the APOE ε4 allele, associated with an elevated risk of late-onset Alzheimer’s disease (APOE ε4 allele represents about 5–30% of the APOE alleles in European populations).

### Influence on professional attitudes

Most participants approached the data from a personal perspective, not a professional. Their focus was mostly on the use of the data as a personal resource — to identify genetic variants that would provide insight into their own present or future health status — without any reference to the professional motivations for participation. However, participants also had various professional reasons for participating and 11/14 indicated in post-test interview that they considered the project useful from a professional perspective (Box 3). The participants were asked if participation in genomic sequencing should always be discussed with family members as well. The replies varied, for example, one participant stated that the decision is always individual, but would recommend discussing with family members. Another participant thought it should be an individual decision similar to a decision to make a doctor’s appointment.
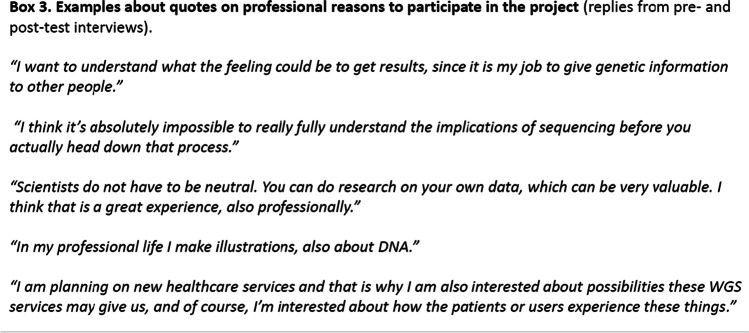


When asked if the doctor should be able to decide which genomic information can be given for patients, one participant replied that people should have control over their data. Another participant said that counselling would be a good idea and thought the person and the counsellor or doctor should decide together.

In Box 4, we present examples of participants’ views relating to relevant issues when planning possible implementation of WGS in health care in general, and the role of genetics clinics in this. The quotes illustrate the vastly different views of the participants. When specifically asked about the influence on their professional attitudes, all participants unanimously referred to the additional insight the experience had provided. Most were not very concrete about how they wanted to use this insight in their professional life, except for one, who planned to write a book on the experience.
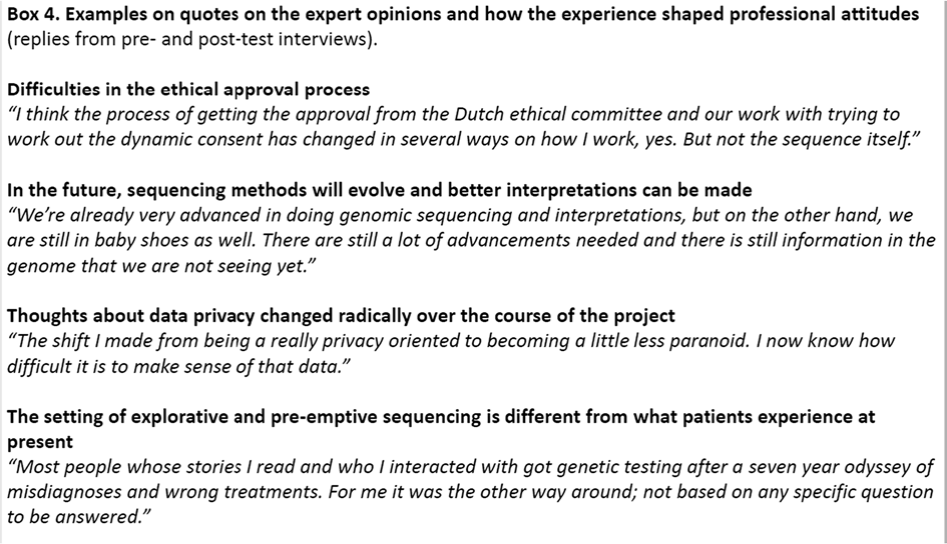


## Discussion

We explored the influence of personal experience of WGS on professional perspectives. We monitored and assessed the expectations for WGS, and to what extent these expectations were met, and identified issues that the genetics regime should be aware of when planning implementation of genome sequencing beyond the diagnosis of rare genetic disorders.

The concept of WGS without any medical indications was new to the local medical research ethics committee (MREC), resulting in a multi-faceted decision process (Appendix 1), finishing with the decision that the study does not fall under the Dutch Medical Research Involving Human Subjects Act.

The participants became aware of the many aspects that require a choice or decision for responsible implementation, far beyond the obvious challenges — privacy, autonomy — that have been discussed extensively. In addition, they came across various challenges that are inherent to the current human genetics ‘regime’, and that may shape the Genetics Clinic of the Future.

First, developing and implementing the appropriate procedures for information and consent for WGS is challenging. Our study proved that there is a great need for dynamic approaches towards counselling, consent and access to data. Individuals progress continuously, and their thoughts, attitudes and preferences change accordingly. This was clearly illustrated by the two participants who decided to withhold from accessing their sequencing data; pregnancy and new insights were important examples for why preferences might change. This change in view was also reflected in those participants that didn’t follow their plan to inform family members or others about their participation or the findings.

Second, examining the data without a specific diagnostic objective was confusing to many participants (Rigter et al. 2014). In clinical genetics, the diagnostic purpose strongly determines the interpretation of sequencing results. The inability of most participants to select which kind of diseases or predispositions they would like to look for clearly reflects how challenging interpretation is without a pre-defined (health related) question. One could see this as an argument for limiting genome sequencing to the traditional clinical context, but we hypothesise that the opposite may be more sustainable; to consider non-clinical genetic findings (and initiate corresponding research) as inspiration for possible innovative new ways to implement genomics beyond traditional clinical genetics (Rigter et al. 2014).

Third, the doctor-patient (or provider-client) relationship may change as a result of personal experience of WGS. The closest comparison is the doctor-who-becomes-patient paradigm, showing that doctors have more empathy and patience with their patients if they are patients themselves, but in genetics the theory has never been tested (Klitzman 2006). However, already in 2015, the European Society of Human Genetics discussed the question: “Should Clinical Geneticists have their Genome Sequenced?” (Santen 2016). The prime argument for doing so was: “Personal understanding of the process that patients go through will improve counseling skills of clinical geneticists” (Santen 2016). Only two of our participants were doctors (a clinical geneticist and a psychiatrist); thus our study did not contribute much to this theory. However, we argue that many other professionals involved in various ways in (genetic) healthcare also have a great impact on the system. The question we should thus ask is: “Should genetics professionals have their genome sequenced?”.

Fourth, the participants varied considerably in prior experience with interpretation of genetic variants. Experienced researchers smoothly extracted their status for clinically relevant variants, (e.g. BRCA1, APOE, or MTHFR). The participants with less experience initially consulted colleagues, family, friends, or experts in the consortium, expressing various degrees of uncertainty. As genetic professionals had difficulties, lay people can be expected to find the interpretation even more difficult. If they will, in the future, often have their genomic data in their possession, appropriate tools and support must be available.

As they entered genomic testing without any prior clinical objective, only four of them were able to indicate the variants they would be most interested to learn about. The other participants were not able to make clear choices on the information they wanted to get out, and were instead guided by the software tools provided to them.

Finally, participants’ opinions varied considerably about who should decide whether to be tested or not, and who should be involved in the process of taking that decision. Many agreed that the individual should be autonomous, but appreciated the idea of clinical support or counselling to assist in decision making. Opinions of the participants about whether the family should be acknowledged in the decision-making process were inconsistent. This seemed to depend on the context; significance of the expected results and relationship with family members.

This is not the first study to focus on genetic testing of healthy individuals, and even the profile of the participants — professionals in genetics-affiliated areas — is not totally new. Yet the focus on the impact of personal experience on professional thinking is unique. Others have captured the personal experience from — supposedly healthy — participants to beyond-the-clinic genome sequencing projects, but the focus has either been on the impact of medically relevant findings, or public awareness of genomics (Ball et al. 2014; Beck et al. 2018; Machini et al. 2019). The difference in the focus is subtle but significant, as the framework for future applications of WGS — e.g. analysis and interpretation tools, consent, legislation — is strongly influenced by experts in genetics. As the results indicate, the perspectives of such professionals on how this framework should be constructed are not similar nor static. Adding a personal experience to the professional view reveals various issues that require more in-depth and diversified consideration.

An interesting result was that the participants shared the results as planned or to a greater extent than planned to colleagues and partners, but less than planned to parents and siblings. The reason might be that they did not consider the results interesting for the relatives if actionable findings were not detected. On the other hand, it might reflect the complexities in informing relatives about shared genetic results (van den Heuvel et al. 2019). This is a challenge that genetics clinics will increasingly face: who will be the messenger in informing of genomic results, who will take the responsibility and who will assure that the data privacy issues are being considered.

This study revealed personal expectations, experiences and individual impact of genome sequencing, and it showed that the decisions, thoughts and emotions relating to WGS are complicated and may change over time. We look forward to similar studies in diverse professional and societal groups to better prepare for future implementation of genomics to healthcare and beyond.

We believe that analysing the experiences of professionals might help those offering WGS to population, whether as part of healthcare, research or DTC business, to understand the type and extent of the essential support.

If genomic testing of healthy individuals becomes a common procedure in healthcare and outside of it, there is a need to thoroughly discuss its position. Studying healthy subjects was not counted as medical research by the MREC. Our study was a research project but if conducted outside of research setting: it could be considered as a part of healthcare, something complementing healthcare or an action better suited outside of healthcare. In the ethical approval process, MREC proposed to guide the participants to appropriate genetic counselling in the country of residence. None of them used this option and so we do not know if the overloaded genetics clinics might have welcomed them.

## Supplementary Information

Below is the link to the electronic supplementary material.

Supplementary file1 (PDF 135 KB)

Supplementary file2 (PDF 105 KB)

Supplementary file3 (PDF 39 KB)

Supplementary file4 (PDF 144 KB)

Supplementary file5 (PDF 157 KB)

Supplementary file6 (PDF 183 KB)
