# Supplementary material for: Simulating the Genetics Clinic of the Future — whether undergoing whole-genome sequencing shapes professional attitudes
Source: J Community Genet. 2022 Jan 27;13(2):247–56. doi: 10.1007/s12687-021-00561-0 (PMC8941039; doi:10.1007/s12687-021-00561-0)
Supplement: Supplementary file 1 — Supplementary file1 (PDF 135 KB) [file 12687_2021_561_MOESM1_ESM.pdf]

Appendix I. Overview of the review process at the Medical Research Ethics Committee.

Date	Action
November 2015	Application to MREC to review the research proposal nr. 15/676, titled “The Genetics Clinic of the Future”.
February 2016	MREC seeks advice from the Central Committee on Research Involving Human Subjects (CCMO)
February 2016	Advice from CCMO to MREC: appoint an ad-hoc committee with relevant experts to evaluate the contents of the proposal
March 2016	First response from MREC; an ad-hoc committee of MREC members with relevant expertise required additional information
March 2016	Response to questions from ad-hoc committee
April 2016	Submission of revised information letter and consent form
May 2016	Confirmation from MREC that the Medical Research Involving Human Subjects Act (WMO) does not apply to the project
