# Supplementary material for: Simulating the Genetics Clinic of the Future — whether undergoing whole-genome sequencing shapes professional attitudes
Source: J Community Genet. 2022 Jan 27;13(2):247–56. doi: 10.1007/s12687-021-00561-0 (PMC8941039; doi:10.1007/s12687-021-00561-0)
Supplement: Supplementary file 2 — Supplementary file2 (PDF 105 KB) [file 12687_2021_561_MOESM2_ESM.pdf]

PARTICIPANT INFORMATION SHEET GCOF SIMULATION PROJECT

This sheet summarises the project and key points that you need to consider before participating in the GCOF Simulation Project. Please ask as many questions as you want and share this information sheet with friends and family.
If you don't want to take part in this project, you don't have to. It's up to you. You don't have to give a reason and your role in the consortium will not be affected if you say no.

ABOUT THE GENETICS CLINIC OF THE FUTURE (GCOF)
The 'Genetics Clinic of the Future' (GCOF) started on January 1 2015 as a 2.5-year research project, granted by the H2020 Framework of the European Commission (HCO15-643349-GCOF). The consortium partners are deeply aware of how much the potential of next-generation sequencing (NGS) for research and diagnostics depends on solutions to major challenges around data sharing and control, informed consent, and the role of genome data within and beyond the clinic. Since these challenges cross disciplinary boundaries (for example simultaneously touching upon genomics, clinical genetics, bioinformatics, ethics, and law), viable solutions will depend on new forms of collaboration among the range of disciplines involved. Within the GCOF project, widely divergent disciplines work closely together to develop five fundamental elements of the genetics clinic of the future:

1. Data control; to map out the data control concept by (1) exploring novel approaches to data access, and (2) involving patients in the design of genome data control mechanisms
2. Consent framework; to define a new approach to consent that supports modern forms of diagnostics data (-omics data, genome sequence, deep phenotypes) and changing diagnostic needs
3. Data sharing across research and diagnostics; to explore novel forms of data exchange between research and patient care in close collaboration with patients
4. Engagement and mutual learning; to create a European infrastructure of 'ambassadors' from various levels of society, who help in engagement of all relevant communities into developing the Genetics Clinic of the Future
5. Policy options; to engage policy makers in the development of the Genetics Clinic of the Future, by (1) developing novel approach to engage policy makers, and (2) assessing the added value of a genetic test

The GCOF Simulation Project

The GCOF Simulation Project is developed in the context of the 'Genetics Clinic of the Future'. It enables the consortium to go beyond a theoretical consideration of the challenges ahead, by gaining firsthand experience of having their genome sequenced. As a partner in the Genetics Clinic of the Future consortium, you can use your own data in considering solutions, scenarios and situations that may occur in the future clinic.

UMCU (Partner 1) has offered whole-genome sequencing to 15 representatives from each consortium partner, having explicitly consented to take part. The participants will receive their own personal genome data, which they can use according to their wishes in the remainder of the project. They will use their own data to explore some of the technological, ethical, legal, economical and political questions they have as an expert in the GCOF project. The detailed conditions and qualifications for participation are part of the consent framework (Appendix I, Chapter 9).

Why have you been invited to participate in the GCOF Simulation Project?
You are a representative of one of the partner organisations in the H2020 'Genetics Clinic of the Future' project. Within your organisation it was decided that you would participate in the GCOF Simulation Project based on voting, group discussion, straw drawing or other forms of selection. You have further been invited because you are assumed to be a healthy, highly educated and well-informed individual.

Key message: You have been offered sequencing of your whole genome as part of the GCOF Simulation Project. The Genetics Clinic of the Future (GCOF) Consortium initiated the GCOF Simulation Project to supplement experts' hypotheses about the future of medical genetics with their personal experience of having their genome sequenced

GENOME SEQUENCING

Your genome is all of the genetic information in your body's instruction manual. Some of your genome is unique to you, but you do share most of it with your relatives. Your genome is made of DNA which is a series of letters which can be looked at, one-by-one, using a technique called sequencing. Recent developments in science and technology mean that it has become possible to look at the whole genome.

What can sequencing a whole genome tell us?
Learning more about the work of your genes could give vital clues about the cause of disease and help to improve treatments. When different whole genome sequences of patients with the same condition are compared, it is possible to see patterns. These patterns can be put together with health information about the patient and many other participants. Once this is done we may be able to link particular patterns with whether people become ill and how severe their illness is likely to be. Sometimes these patterns can also give clues as to the best treatments for a patient, or may reveal information that could be important to other family members.
In order to distinguish these disease-associated variants from genetic variation that is not disease-related (often referred to as 'normal genetic variation'), it is important to read not only the genomes of patients, but also those of healthy individuals. Once you have your personal genome data at hand, you could be one of those individuals.

What does participation entail?
After you have read and considered this information sheet, you will be asked to:

1. Add your contact details to the consent form, and date and sign it if you want to join the Project
2. Donate a sample of saliva using the collection kit that will be sent to you
3. Send the sample along with the dated and signed consent form back to UMC Utrecht using the return envelop.
4. Agree that authorised staff from this Project may contact you directly after sequencing to arrange data hand-over
5. Agree that authorised staff from this Project may contact you in the future for invitations to follow-up research projects (for which you will subsequently asked to provide separate consent)

What will happen to your sample after you have sent it to UMC Utrecht?
1. We will use your samples to extract your DNA so we can undertake whole genome sequencing (looking at all of the genetic code). This will take place at the national sequencing facilities of the Hartwig Foundation in the Netherlands
2. Instead of your name being used to identify your samples and data, they will be labelled with a unique code number, which means you are not directly identifiable.
3. Once the DNA sequence has been obtained from your sample, the sequence data is sent electronically to the bioinformatics team for analysis. The team will perform primary analysis, and generate a file with uninterpreted genetic variants that are present in your genome compared to the reference genome. All analysis files (of type *.fastq, *.bam and *.vcf) will be copied to an encrypted external hard drive, and **deleted from the sequencing servers**
4. Usually, not all your sample is used up for sequencing. All the remaining DNA and any other samples we take will be securely stored by UMC Utrecht until you receive your personal genome data.
5. All data generated from your sample - as well as your sample itself - will be destroyed once you receive the external hard disk containing your personal genome information
6. There may be certain circumstances where your DNA will not be sequenced. Should this occur the explanation will be given to you by the coordinator
7. If you wish to further analyse the data yourself, you will receive the necessary support to do so from the consortium partners
8. You will be asked to share your experience with the consortium, in a format that you feel comfortable with

Key message: Your genome will be sequenced. You will receive uninterpreted personal genome data on an external hard disk.

Data and information

Who will have access to your data?
The bioinformatics team of UMC Utrecht will perform the primary analyses. Therefore they are the only people who require access to your data, specifically with the purpose to perform primary analyses, and storage on an external hard disk. After re-

ception of the hard disk, you are the only person who decides about further access to your data.

Can you access your data?
You are the owner of your data, so yes, you can. However, you may require support to turn your data into a readable format, or to perform further interpretation. You can put a request for such support with one of the consortium partners, or search for it outside the consortium. The consortium will provide central facilities for further analyses and interpretation of your data. If you decide to make use of these facilities, you will be asked to provide separate consent. The facilities will be available at no charge. Alternatively you can look for support in further analyses and interpretation of your data outside the consortium.

How can we assure your information remains secure and confidential?

UMC Utrecht can only assure security and confidentiality during the process of sequencing and primary analysis. During that process:
- your data will be on a controlled access server in the Netherlands, whose security level meets national and international data standards
- your name and other personal details are held separately from the data. Your data is identified only by a code.

From the moment you receive the external hard disk containing your personal genome information, UMC Utrecht can no longer guarantee secure and confidential use of your data. UMC Utrecht will thus transfer liability to you from that moment on, as agreed in the consent form.

Will this affect my insurance?

Any medical treatment you have may need to be disclosed to an insurer who wants it, however:
- Under European law, the results of your whole-genome sequencing carried out in the GCOF Simulation Project are not disclosable to insurers. This is because they are part of a healthcare and research project
- you do not therefore have to disclose to an insurer that you are part of the GCOF Simulation Project now or in the future or reveal the results of any tests returned from the Project

What might we contact you about in the future?
As a partner in the consortium, you are expected to contribute to the consortium discussions about the simulation project. We ask you to donate as much information about yourself and your genome as you feel conformable with.

Individual consortium partners will invite you to contribute research in the context the ‘Genetics Clinic of the Future’ Project. We ask that you are willing to be contacted for that purpose. This might include ethically approved trials of new medicines or re-

search about your views on aspects of the Project. You are only agreeing to be asked. If you don't want to take part in the research when it is suggested then you don't have to. The partners may need further samples or specific information from you but if they want this, they will ask your permission. You don't have to say yes.

Key message: You own and control your personal genome data. You have access to tools to further analyse and interpret your data, but you are in no way obliged to do so. Consortium partners may invite you to participate in research in the context of the 'Genetics Clinic of the Future'.

COUNSELING AND SUPPORT

Will you receive counseling?

You will not be sequenced as part of a medical examination, and thus you will not be entitled to formal genetic counseling, although that may depend on legislation and guidelines in your country. Whereas the consortium does not provide centrally organised counseling, it will be able to provide you with the support you need. Depending on your preferences, the coordinator and other participants may help you in identifying appropriate counsellor in your region, or may be able to give you comprehensive information and answer your questions collectively.

What are your options for further analysis and interpretation?

If you have the required tools available, you are entitled to further analyse your data yourself. Alternatively, partners within the consortium have tools available for further analysis. These tools do not only include genome analysis software, but also clinical interpretation knowledge and skills. These tools are freely available during the Project, and are subject to partner-specific terms of service.

Key message: If you want, there are many options to carefully further analyse and interpret your data

BENEFITS AND RISKS

What are the benefits of joining the GCOF Simulation Project?

Your research in the context of the 'Genetics Clinic of the Future' project will be significantly enriched with the personal genome sequencing experience of you and other member of the consortium. That is the primary benefit of participating in the Project.

Depending on how you further use your data, you may benefit in terms of your healthcare personally but you may not benefit personally at all. Other individuals may benefit from the knowledge generated by this Project

What are the risks of joining the GCOF Simulation Project?

The only immediate risks of doing the Project is that you will be identified as someone taking part in the Project. Whereas the consortium will not disclose any names or

contact details of participants to the GCOF Simulation Project, your name may already be in documentation on the 'Genetics Clinic of the Future' Project. Also, the other participants can deduce your name from mailings and discussions in the context of the GCOF Simulation Project. We will always do everything we can to prevent any (unintended) violation of your privacy, but you must be aware that the risk is substantial.

There might also be new ways in the future to link information back to you directly, but we will keep up with these developments in order to inform you on optimal protection of your data throughout the Project. We believe that the likely benefits of taking part in the project for participants outweigh this remote risk.

How to withdraw from the project
If you want to leave the Project after you have joined, you are able to withdraw by asking the coordinator for a withdrawal form, completing this form and returning it to them. You can only opt for withdrawal from the GCOF Simulation Project. You will then not be contacted anymore for further requests in the context of the GCOF SIMULATION Project. You will not be excluded from any meetings or discussion in relation to the GCOF Simulation Project, and you will remain a member of the consortium

Key message: You will likely only benefit from the Project as a researcher, not as a citizen. You may be identified. You can withdraw.

The consortium members will be happy to answer as many questions as you want. Please share this leaflet with friends and family if you would like to.

Thank you for considering taking part in the GCOF Simulation Project.
