# Supplementary material for: Simulating the Genetics Clinic of the Future — whether undergoing whole-genome sequencing shapes professional attitudes
Source: J Community Genet. 2022 Jan 27;13(2):247–56. doi: 10.1007/s12687-021-00561-0 (PMC8941039; doi:10.1007/s12687-021-00561-0)
Supplement: Supplementary file 3 — Supplementary file3 (PDF 39 KB) [file 12687_2021_561_MOESM3_ESM.pdf]

CONSENT FORM WHOLE-GENOME SEQUENCING

Hereby I give permission for whole genome sequencing (WGS) of DNA derived from my saliva in the context of the Genetics Clinic of the Future Simulation Project.

Sample number:
Name:
Date of birth:
E-mail:

I understand that my participation in the Simulation Project is entirely voluntary and that my future involvement in the GCOF project will not be affected if for any reason I decide not to take part.

The Simulation Project will enable members of the GCOF consortium to explore different aspects of genome sequencing to help anticipate what challenges we might face in the future. I agree to take part in this Project and to have my genome sequenced by UMC Utrecht.

I agree to provide a sample of blood or saliva for genome sequencing, and understand that information about me will be stored securely by UMC Utrecht as part of the sequencing process.

I agree for UMC Utrecht to sequence my genome, following the specifications outlined in the Participant Information Sheet.

I understand that all information about me held by the Project will be treated as confidential. I understand that information from my samples, records or other information I give to the Project will only be accessible in a form which protects my identity.

I understand that UMC Utrecht will only perform preliminary analysis on my genome sequence, and that I **will not** receive comprehensive analyses outlining different aspects of my genome that may have implications for my current and future health.

I agree for UMC Utrecht to provide me with the raw sequencing data on a hard drive, which will be delivered by hand by a member of the research team

I understand that as soon as I have received the hard drive containing my genome sequence data, UMC Utrecht will destroy the data stored on their server and will not keep a backup.

I understand that as soon as I have received the hard drive containing my genome sequence data, my blood or saliva sample will be destroyed by UMC Utrecht.

I understand that as soon as I have received the hard drive containing my genome sequencing data, and the original copy has been destroyed, UMC Utrecht will relinquish any responsibility for security, analysis or further use of this data.

I understand that future use and analyses of this data is my responsibility, and that although I can seek advice from other members of the GCOF consortium, the GCOF

consortium, funding body and anyone associated with the project will not be liable for any information I discover that may cause distress or concern relating to my current or future health.

I understand that the intention of the simulation project is to explore different scenarios that might arise in the Genetic Clinic of the Future. I agree to the GCOF project to invite me to participate in these simulation exercises using my genome sequence, and understand that future involvement is entirely voluntary and I can withdraw at any time.

Place

Date

Signature
