# Supplementary material for: Simulating the Genetics Clinic of the Future — whether undergoing whole-genome sequencing shapes professional attitudes
Source: J Community Genet. 2022 Jan 27;13(2):247–56. doi: 10.1007/s12687-021-00561-0 (PMC8941039; doi:10.1007/s12687-021-00561-0)
Supplement: Supplementary file 4 — Supplementary file4 (PDF 144 KB) [file 12687_2021_561_MOESM4_ESM.pdf]

Appendix IV. Pre-test interview guide.

Background information

1. Subject details
   - Name
   - Subject id
   - Year of birth
2. Do you have children?
3. Education (select the highest degree)
4. Work

Participation in this study

5. Could you explain why you decided to join this simulation project?
6. Do you think the project is going to benefit your personal life somehow?
7. Do you think the project is going to benefit your professional life somehow?
8. Did you have any doubts in participating to the project?
9. What made you doubt participation?
10. Who did you tell about your participation in the GCOF project?

Health

11. Do you feel that your health condition is good? Rate from 1 (very healthy) to 5 (not healthy at all)
12. Are you worried about your health?
13. Do you think this genetic information might change your health behavior?
14. Are you afraid of worrying results?

Expectations relating to analysis and results

15. Do you think you will need a help of an expert (for example, bioinformatician) to interpret the data?
16. Which analysis tools do you think you are going to use?
17. Based on your genome data, which information are you trying to figure out?
18. Can you list, which information do you wait the most?
19. Do you think your genomic results are going to play a major role your life in the future?
20. Do you plan to see a doctor or bioinformatician if some worrying results appear?
21. With whom are you planning to share the genomic results?

Society and Future

22. Do you think everyone should have the opportunity to have their genomic data for free?
23. Do you think everyone should be sequenced for research to benefit the society?
24. Do you think everyone who is sequenced should give researchers access to their data to enhance research and

thus benefit society?
