# Supplementary material for: Simulating the Genetics Clinic of the Future — whether undergoing whole-genome sequencing shapes professional attitudes
Source: J Community Genet. 2022 Jan 27;13(2):247–56. doi: 10.1007/s12687-021-00561-0 (PMC8941039; doi:10.1007/s12687-021-00561-0)
Supplement: Supplementary file 5 — Supplementary file5 (PDF 157 KB) [file 12687_2021_561_MOESM5_ESM.pdf]

Appendix V. Post-test interview guide.

Background information

1. Name or Subject id
2. Since the pre-test interview: Has there been major changes in your life relating to your family status, health or employment?

Receiving the hard drive

3. How did you feel when you received the hard drive containing your genomic data?
4. Did you look at the files on the hard drive?
5. How long after you received your hard drive did you plug it in your computer?
6. Did the file format meet your expectations? Please explain.
7. Will you continue on going through your data after the project ends?

Privacy issues/ Data control

8. Have you had thoughts about the possibility that someone might look in to your data without your permission?
9. How well do you store your data?
10. To whom have you told about your participation in the GCOF project?
11. With whom did you share the genomic results?

Analysis

12. Based on your genome data, which information did you figure out? (you can select multiple)
13. Were you able to interpret your data before getting help from Bio.logis? If so, how?
14. (no, I analyzed the data by myself) Which analysis tools did you use?
15. (no, I did not interpret the data) Do you believe you will look at the results at some point in the coming months/years?
16. (no, I did not interpret the data) Does it bother you that there are results possibly telling about your future health but you don't know them?
17. Did you feel that the results were clear and you could comprehend them or that there was a myriad of results and most of it was very unclear/difficult for you/for those helping you to interpret or something in between?

Health

18. Do you feel that your health condition is good? Rate from 1 to 5.

19. Are you worried about your health?
20. Did the information change your health behavior?

Analysing one's results

21. Did you go to see a doctor because of the information you received from the project?
22. Do you feel it OK that you yourself decided about this genomic sequencing? Or would you rather recommend that there always would be a MD prescribing such analyses? Or would you recommend that it would be asked from the family members as well?
23. Did you receive some worrying results?
24. (Yes, I received worrying information) Would you like to describe in a few words what was worrying?
25. Do you feel that some part of the project and/or some results have somehow affected your life?
26. Do you trust your genomic results and interpretation what the results might mean?
27. Do you feel that it would have been helpful to meet a genetic counselor or a clinical geneticist after getting your results, for example, to discuss which results would be useful to search from the data?
28. If you could go back to that moment when you decided to participate in the project, what would you do?
29. Do you think you were well informed on the possible outcomes of the project?
30. Do you think you were well informed on the possible risk of the project?
31. Are you satisfied with your decision to participate in the project?
32. Would you recommend to your friends and family to get their genome sequenced?
33. Which were the most interesting parts of the results; can you list top 5 variants?
34. Considering your daily life, do you think the project useful?

Work-related questions, professional/expert approach

35. Considering your professional life, do you think the project useful?
36. Did the experience of undergoing whole genome analysis affect the way you think about your work?
37. Did it change the decisions you made in your work?
38. How did you experience 'exposure' to other disciplines? Were you able to have a meaningful discussion? Can you reach a level where it matters to your own work?

Society and future

39. Did you enter into a public or private healthcare/clinic with your genome data? If yes, what was the practice? Did the doctor take the results into account, etc.?
40. If the patients/citizens in the future get their WGS (as you just did): should somebody (the doctor?) restrict the data so that, for instance variants leading to neurological diseases that do not have a cure

would be hidden from the results? Or should the patients/citizens have full authority to see all results which they choose to see?

41. If in the future, patients and citizens in general are often offered the opportunity to have their full genome sequenced, how would you advise the healthcare/the company marketing the test to inform the participants beforehand? Please list some points that you find important.
42. It has been predicted that in the future research and clinic will co-operate more. Do you believe that individual results from biobanks can benefit the health of the participants?
