# Supplementary material for: Simulating the Genetics Clinic of the Future — whether undergoing whole-genome sequencing shapes professional attitudes
Source: J Community Genet. 2022 Jan 27;13(2):247–56. doi: 10.1007/s12687-021-00561-0 (PMC8941039; doi:10.1007/s12687-021-00561-0)
Supplement: Supplementary file 6 — Supplementary file6 (PDF 183 KB) [file 12687_2021_561_MOESM6_ESM.pdf]

Appendix VI. Questionnaire.

1. Subject number
2. The second interview took place about 3 months after you had received your data. Have you returned to your data or results after that time?

(questions for participants, who had returned to their data/results)

3. Why did you return to your data? (If you wish, please explain)
4. To which results exactly have you returned to? Your raw genomic data, Bio.logis report or other? Please, share:
5. Have you been worried because of the results you received? (If you wish, please explain)
6. Did the information change your health behavior somehow? (If you wish, please explain)
7. In the second interview we enquired whether you had shared your genomic results with anyone. Now we would like to know if you (further) shared the results with others?
8. Which information did you find the most interesting parts of the results?
9. Has your involvement in the simulation project changed your views when working in genetics?
10. Do you still keep your genomic data stored somewhere? Or have you deleted it?

(questions for participants, who did not return to their data/results)

11. You replied that you did not return to your genomic results. What was the reason?
12. Has your involvement in the simulation project changed your views when working in genetics?
13. Do you still keep your genomic data stored somewhere? Or have you deleted it?

(to all respondents)

14. Would you like to add something?
